# Let's have another cup of green tea!

**DOI:** 10.1016/j.heliyon.2025.e42651

**Published:** 2025-02-15

**Authors:** Benno F. Zimmermann, Lilli Drees

**Affiliations:** University of Bonn, Faculty of Agricultural, Nutritional and Engineering Sciences, Institute of Nutritional and Food Sciences, Käthe-Kümmel-Str. 1, 53115, Bonn, Germany

**Keywords:** Green tea, Flavanols, Catechins, Theanine, Caffeine, Second infusion

## Abstract

For this study, the content of the main flavanols, theanine and caffeine in 17 samples of loose leaf green tea were analyzed regarding the leaves as well as the first and second infusions. The infusion conditions followed the instructions on the labels as consumers are supposed to do. The leaf green teas were infused twice and the infusions were examined separately. The galloylated flavanols, *i.e.*, epicatechin gallate and epigallocatechin gallate, were found in roughly equal concentrations in the first and second infusions (medians of 96 % and 106 %, respectively, relative to the first infusion). The non-galloylated flavanols, *i.e.*, epigallocatechin and epicatechin, and caffeine had mostly lower concentrations in the second infusion down to two thirds compared to the first infusion (medians of 72 %, 73 % and 83 %, respectively). Theanine is always higher in the first infusion, in some cases two times higher (median of 58 %). This correlates with the polarity of the compounds. It can be concluded that the second infusion of leaf green tea still contains considerable amounts of caffeine, theanine and flavanols.

## 1 Introduction

Green tea is part of the cultural identity in the producing countries or regions like China and Japan. In the Western world, green tea has gained a lot of attention due to its possibly health-relevant components like theanine and flavanols, especially epigallocatechin gallate (EGCg). It must not be overlooked that caffeine is the only compound in tea with unambiguously recognized positive health effects at defined doses [1]. And last but not least: tea is a hedonistic beverage with a characteristic taste favored by many (and disliked by some).

It is common to infuse the same portion of green tea leaves more than once. In traditional tea ceremonies, the preparation of multiple infusions follows distinct protocols [[2], [3], [4]]. A convenient Western style everyday preparation is to pour a second volume of hot water to the wet leaves from the first infusion. The taste of the second infusion has been described as lighter, less astringent and less bitter [2]. The details of the second infusion, *i.e.*, water temperature and steeping time, are discussed in non-scientific press and social media. The only consensus is: there is no right or wrong. It depends on the tea quality and one's personal preference: it's a matter of taste.

The other question is: does the second infusion still contain relevant amounts of caffeine and possibly health-related compounds like flavanols and theanine? The scientific literature does not answer this question soundly. Yang et al. present data about the flavanols, gallic acid and caffeine in eight consecutive infusions of 30 s at 70, 85 and 100 °C of a specially produced green tea [4]. The temperatures of 70 and 85 °C are common in household green tea preparation, but 30 s of steeping time is rather short. At 70 °C, the second infusions have roughly twice as high concentrations of the analyzed compounds compared to the first infusion. At 85 °C, the second infusion has about 20 % less than the first infusion. Unfortunately, the graphic showing the data doesn't allow for reading exact numbers. However, the short steeping time doesn't reflect common tea preparation. Tea preparation in Sharma et al. is realistic: 3 min at 80 °C [2]. A specially produced green tea was used. The first and second infusion have virtually the same concentrations of gallic acid, main flavanols and caffeine. Two further studies considering the second infusion of green tea did not analyze individual compounds but antioxidant capacity [5,6], which doesn't correspond to health effects or food quality [7].

The aim of this work is to find out whether an average green tea consumer can expect considerable amounts of health-related compounds in the second infusion. We therefore analyzed 17 different medium-level green leaf teas available on the German market. The total amount of theanine, caffeine and the four main flavanols epigallocatechin gallate (EGCg), epicatechin gallate (ECg), epigallocatechin (EGC) and epicatechin (EC) in the leaves and their concentrations in the first and second infusion was determined. All teas were prepared according to the recommendations on their labels.

## 2 Material and methods

### 2.1 Samples

All teas are distributed by TeeGschwendner (Meckenheim, Germany) and are available in Germany and abroad. The samples were kindly gifted by TeeGschwendner. Details about the green leaf teas, including the recommended steeping temperatures and times are compiled in Table 1. All samples are loose green leaf teas with a larger leaf size than in common bag teas.

**Table 1:** Details of the green leaf tea samples as given by the distributor.

Product	Number	Lot	Origin	Steeping temperature^a^	Steeping time^a^	Tea leaf to water ratio^a^	Additional Info
Darjeeling GFTGFOP1 Green Elephant	293	1425421	Darjeeling	85 °C	2 min	11 g/L	
Nepal Himalya View	310	1424697	Eastern Nepal	90 °C	1.5 min	11 g/L	
Nepal Ilam Mao Feng	313	1414788	Ilam region, Nepal	80 °C	1.5 min	12 g/L	
Green Manjolai	355	1425241	Nilgiri, Southern India	90 °C	1.5 min	11 g/L	
China Gunpowder Temple of Heaven	510	1425423	China	75 °C	2 min	11 g/L	Rolled like gunpowder tea
China Mao Feng	516	1414789	China	70 °C	2 min	12 g/L	
Südkorea Seogwang	590	1422312	South Korea	80 °C	2 min	12 g/L	
Südorea Seogwang Sencha	591	1426465	South Korea	80 °C	2 min	12 g/L	
Myanmar Green Pindaya Bio	596	1425103	Myanmar	75 °C	2 min	11 g/L	
Grüntee Kampagne	597	1426035	Yunnan, China	90 °C	1.5 min	12 g/L	
Neuseeland Zealong Green	665	1417700	New Zealand	80 °C	2 min	13 g/L	
Japan Sencha	700	1425104	Japan	90 °C	1 min	12 g/L	
Japan Bancha	701	1426476	Japan	80 °C	2 min	11 g/L	Late harvest
Japan Sencha Extra Fine	705	1426615	Japan	70 °C	2 min	12 g/L	
Japan Gyokuro	720	40952/20	Japan	60 °C	2 min	11 g/L	Fully shaded
Japan Shincha Kirisakura First Flush	723	40953/50	Japan	60 °C	2 min	11 g/L	Early harvest, 14–20 days shaded, cultivar Saemidori
Rainforest Rescue Tea	865	1425102	Indonesia	90 °C	2 min	12 g/L	Green tea, but shaped like oolong

a As recommended on the label, to get a tasty brew.

### 2.2 Tea preparation

The tea infusions were prepared according to a previously published protocol [8] with slight modifications. The recommended amount of tea leaves (see Table 1) was weighed in a stainless steel tea sieve (3139, TeeGschwendner). The tea sieve was put in a porcelain mug (Waters, Eschborn, Germany) and the mug with the sieve and leaves was put on a balance. 250 g of hot tap water (moderately hard: magnesium 5–8 mg/L, calcium 24–38,6 mg/L; according to Stadtwerke Bonn, the local public water supplier) was added. The exact weight of the water was documented and converted to volume using the specific mass. After half of the steeping time and shortly before the end, the tea leaves were whirled up by moving the sieve up and down. At completion of the steeping time, about 1 mL of the infusion was withdrawn using a syringe and immediately pushed through a membrane filter (ChromafilPET, diameter 15 mm, pore size 0.45 μm; Macherey + Nagel, Düren, Germany) into a vial to allow fast cooling to room temperature. The sieve with the tea leaves was taken out of the infusion and allowed to drip off.

The tea was injected directly into the HPLC without further treatment for analysis of theanine. For analysis of the flavanols and caffeine, 100 μL of the filtered tea infusion was diluted with 455 μL of stabilization solution (acetonitrile + water, 1 + 9, with 500 mg ascorbic acid/L and 5 mg EDTA/L) and 40 μL ethanol and analyzed by HPLC within 24 h. The mug was emptied and put back on the balance. For the second infusion, hot water was added until the balance showed 250 g; this takes the adhering water into account. The following steps were the same as before. Time and temperature of both infusions were the same and as indicated on the labels (see Table 1). All infusions were prepared in duplicate or quadruplicate (see Supplementary data).

### 2.3 Exhaustive tea extractions

#### 2.3.1 Exhaustive extraction of flavanols

For determination of the total percentage of flavanols in the tea leaves, the tea leaves were ground in a ball mill (MM 2000, Retsch, Haan, Germany) and then extracted as described before [9]. Briefly: 40 mg of the powder was extracted with 10 mL ethanol + water, 70 + 30, for 10 min in an ultrasonic bath. A portion of 1 mL of the extract was pushed through a membrane filter (ChromafilPET, diameter 15 mm, pore size 0.45 μm; Macherey + Nagel, Düren, Germany). 100 μL were diluted with 900 μL stabilization solution (see 2.2) and analyzed by HPLC within 24 h. All samples were extracted in duplicate.

#### 2.3.2 Exhaustive extraction of theanine

For the determination of the total percentage of theanine in the tea leaves, 60 mg of powdered tea was weighed in a 5-mL-volumetric flask and 4.8 mL of water was added. The mixture was vortexed and put in an ultrasonic bath for 10 min. The volume was made up to 5 mL, membrane-filtered (ChromafilPET, diameter 15 mm, pore size 0.45 μm; Macherey + Nagel, Düren, Germany) and analyzed by HPLC. All samples were extracted in duplicate. For the determination of recovery, this method was compared to a four-step extraction with the same conditions. The results of the two extraction methods were not significantly different; therefore, recovery is considered 100 %.

### 2.4 HPLC analyses

The Agilent 1100 HPLC was the same as published earlier [10].

#### 2.4.1 HPLC-UV analysis of flavanols and caffeine

The HPLC parameters were as previously published [8] with modifications: another column was used (XSelect CSH C18, 3 mm × 150 mm, 3.5 μm, Waters, Eschborn, Germany, with a Universal-RP guard column Macherey + Nagel, Düren, Germany) resulting in enhanced peak symmetry and better resolution. The gradient of the eluents A (acetonitrile with 0.1 % formic acid) and B (water with 0.1 % formic acid) was as follows: 0 min, 7 % A; 10.5 min, 22.6 % A; 11.5 min, 95 % A; 13.5 min, 95 % A; 14.5 min, 7 % A; 18.0 min, 7 % A at a flow rate of 0.7 mL/min. Maximum pressure during a run was around 300 bar. Injection volume was 2 μL. Detection wavelength was 278 nm.

The compounds were identified by comparison with authentic reference compounds: EGCg (Merck, Sigma-Aldrich, Taufkirchen, Germany), EC (Sigma Chemical Co, St. Louis, USA), ECg and EGC (Biorbyt via Biozol, Eiching, Germany), caffeine (Thermo Fisher, Kandel, Germany). The same compounds were used for external calibration.

#### 2.4.2 HPLC-UV analysis of theanine

An Atlantis T3 C18 column (3 mm × 150 mm, 3 μm with an Acquity UPLC BEH C18 guard column, Waters, Eschborn, Germany) showed a stronger retention for theanine. With 100 % B (water with 0.1 % formic acid) as eluent at a flow of 0.7 mL/min, theanine had a retention time of 2.1 min. After that, the eluent composition was changed to 95 % A (acetonitrile with 0.1 % formic acid) within 5 min and maintained for further 2 min to wash all other compounds off the column. Within 1 min, eluent composition was brought back to 100 % B and the column was re-equilibrated for 3 min. Maximum pressure during a run was around 280 bar. Injection volume was 2 μL. Detection wavelength was 210 nm. Theanine was identified by comparison with the authentic reference compound, that was kindly gifted by Taiyo GmbH (Gevelsberg, Germany). The same compound was used for external calibration.

## 3 Results and discussion

### 3.1 Flavanols, theanine and caffeine in the first and second tea infusions

**Fig. 1:**
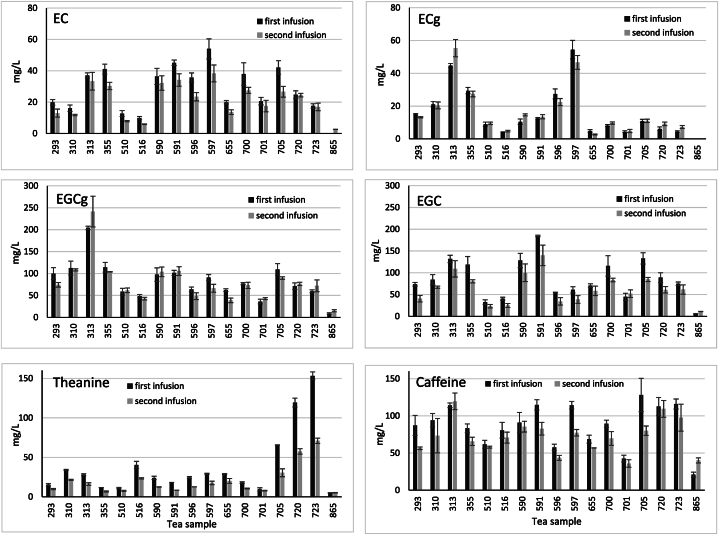
Concentration of the phytochemicals in the first and second infusions.

The concentration of flavanols, theanine and caffeine in the first and second tea infusions is shown in Fig. 1. In the Supplementary data the numerical values are given. Among these numbers, the most interesting fact is that in the second infusion the concentrations of the flavanols and caffeine are similar to those in the first infusion with some tendencies and exceptions.

The concentrations of flavanols, theanine and caffeine in the first infusion are in accordance with previously published results regarding flavanols and caffeine [5,8,11,12] and regarding theanine [13,14]. The wide range of the published results is explained by the differences in the tea leaves and also by the different brewing methods. It is known that tea leaf particles are extracted faster when free-floating compared to bag tea and that stirring speeds up extraction [15].

The analyzed compounds can be divided into two groups regarding the percentage in the second infusion relative to the first infusions:

1 The group of equal distribution: the galloylated flavanols EGCg and ECg are present in the second infusion in portions of 96 % and 106 % (medians), respectively, relative to the first infusion.
2 The group of preferred first infusion: the non-galloylated flavanols EGC and EC are present in the second infusion in portions of 72 % and 73 % (medians), respectively, compared to the first infusion. Caffeine behaves similarly with a median of 83 %. The lowest percentage in the second infusion is observed for theanine with a median of 58 %.

This grouping can be explained by the different polarity of the compounds: theanine is the most polar compound making it easier to extract. The galloyl moiety of EGCg and ECg could lead to the hypothesis that these compounds are more polar than the non-galloylated flavanols. But the latter are eluted earlier in RP-HPLC indicating their higher polarity. Therefore, EGC and EC are mostly found in higher concentrations in the first infusion while EGCg and ECg are more equally distributed in both infusions. This confirms previous results regarding simple infusion and extraction on an industrial scale [[16], [17], [18]].

EGCg, the most discussed putative health promoter, is found in the second infusions in similar (at least 95 %) or higher concentrations relative to the first infusion in nine out of 17 samples of this study. But even the lowest concentration in the second infusion is 63 % compared to the first infusion. Caffeine tends more to the first infusion: just three samples have more than 95 % in the second infusion. The lowest percentage in the second infusion is 62 % compared to the first infusion, which is still considered a substantial contribution to daily caffeine intake. The other highly discussed compound, theanine, is found in only one second infusion at 95 % or more compared to the first infusion, namely 103 % in sample 865. The lowest value is 46 %.

In all cases, a wide range of results is found. This is due to the different properties of the tea leaves and the different preparation conditions. The leaf size obviously plays a role in the case of tea 865, which is the only tea with entire leaves that are moreover rolled into compact balls. During the first infusion, they unfold and are easier to extract in the second infusion. This correlation between size and extractability has been described before [5,9,15,19]. Therefore, tea 865 shows the highest percentages in the second infusion and the smallest absolute concentrations although the phytochemical content in the leaves of this tea is not particulary low. Thus, it is hypothesized that smaller leaf particle sizes, like often used in simple quality tea bags for example, lead to lower percentages in the second infusion because the first infusion already extracts a greater portion of the compounds.

Sharma et al. found equal or almost equal concentrations of the flavanols in the second infusion of one tea. The caffeine concentration in the second infusion was at 112 % compared to the first infusion [2]. Yang et al. chose a steeping time of just 30 s and the tea was ground to 1–2 mm particles. Thus, the smaller particles may compensate for the shorter steeping time. At 70 °C, the second infusion has about double the concentration of the flavanols and caffeine, as far as identifiable in the graphic in that paper. At 85 °C, the first infusion is in advantage by max. 20 % more than the second infusion. Overall, our data confirms the pioneering findings on two teas in the two earlier studies regarding flavanols and caffeine.

### 3.2 Extractability of flavanols, theanine and caffeine in the first and second tea infusions

The differences of absolute concentrations in the infusions are caused by the combination of extractability and the content of the compounds in the tea leaves. The contents of flavanols and theanine in the tea leaves are shown in Table 2. The contents are in accordance with other published values regarding flavanols [8,11,[20], [21], [22], [23]] and regarding theanine [21,[24], [25], [26], [27]].

**Table 2:** Content of phytochemicals in the tea leaves and extraction efficiencies. All values are means (n = 2 or 4) ± standard deviation.

**Tea sample**	EGC			EC			EGCg			ECg			Theanine		
	Content in the tea leaves [mg/g]	Extraction efficiency of the first infusion [%]	Extraction efficiency of the second infusion [%]	Content in the tea leaves [mg/g]	Extraction efficiency of first the infusion [%]	Extraction efficiency of the second infusion [%]	Content in the tea leaves [mg/g]	Extraction efficiency of the first infusion [%]	Extraction efficiency of the second infusion [%]	Content in the tea leaves [mg/g]	Extraction efficiency of first infusion [%]	Extraction efficiency of the second infusion [%]	Content in the tea leaves [mg/g]	Extraction efficiency of the first infusion [%]	Extraction efficiency of the second infusion [%]
**293**	25.81	25.53	19.1	8.40	21.17	17,8	66.09	13.50	11,8	7.75	17.28	18,8	4.65	29.46	27,4
	±2.45	±2.66	±3.7	±0.73	±2.42	±3.5	±5.14	±1.95	±1.1	±0.62	±1.39	±1.5	±0.02	±3.12	±2,6
**310**	27.74	27.21	30.1	5.66	25.34	25,4	85.80	11.64	13,0	16.42	11.47	12,8	7.82	39.39	41,4
	±0.86	±3.61	±1.3	±0.25	±3.29	±1.4	±2.05	±1.58	±0.4	±0.38	±0.85	±1,3	±0.04	±1.12	±12,7
**313**	33.14	34.01	41.5	10.22	30.70	39,3	101.40	17.05	23,9	22.19	17.16	25,1	4.47	52.70	63,7
	±0.90	±3.13	±7.4	±0.29	±1.26	±6.9	±4.20	±0.82	±3.7	±0.85	±1.09	±2,7	±0.01	±2.47	±4,4
**355**	32.90	32.73	33.0	12.39	30.08	31,8	79.05	13.11	13,8	11.02	24.31	29,9	2.38	44.48	46,5
	±0.87	±4.98	±1.7	±0.83	±2.74	±3.2	±2.80	±1.16	±0.5	±0.22	±1.74	±1,9	±0.03	±0.73	±4,9
**510**	21.29	13.32	11,4	4.54	25.19	21,1	70.01	7.53	8,7	6.63	12.29	14,8	2.73	36.02	39,3
	±0.64	±2.50	±2.3	±0.38	±4.36	±1.8	±2.01	±1.07	±1.1	±0.14	±1.64	±0,8	±0.04	±2.76	±3,2
**516**	18.26	18.20	13,8	2.88	27.80	23,7	83.64	4.75	4,5	6.87	4.87	6,1	10.2	33.19	28,6
	±0.58	±1.92	±2.5	±0.03	±2.56	±0.7	±1.97	±0.32	±0.4	±0.14	±0.16	±0,7	±0.2	±4.12	±1,3
**590**	37.48	30.57	32,0	9.63	31.35	40,6	61.71	13.10	16,1	5.25	16.58	27,9	3.04	64,62	96,7
	±0.28	±5.02	±6.1	±0.06	±4.70	±5.3	±0.04	±2.21	±1.5	±0.01	±2.50	±0,8	±0.03	±5.41	±1,5
**591**	38.00	39.99	51,1	9.29	40.01	51,1	54.41	15.35	19,1	4.72	22.02	30,6	2.18	69.07	103,8
	±1.27	±1.42	±8.3	±0.49	±2.56	±6.6	±0.58	±0.89	±1.8	±0.16	±1.14	±3,1	±0.04	±1.35	±2,7
**596**	26.72	18.53	14,3	19.58	16.49	13,0	63.11	9.17	7,6	15.78	15.76	15,3	7.75	28.79	20,8
	±2.43	±1.72	±3.7	±1.62	±1.87	±1.7	±6.18	±1.13	±1.4	±1.33	±2.10	±1,9	±0.01	±1.06	±0,4
**597**	18.35	27.37	24,1	15.66	28.50	28,5	53.67	14.01	12,0	16.36	27.57	32,8	5.78	41.62	43,4
	±0.05	±3.50	±6.3	±0.22	±3.67	±4.9	±2.26	±1.30	±2.1	±0.23	±3.13	±4,0	±0.16	±1.72	±5,0
**665**	54.37	13.60	9,5	11.92	12.87	10,1	75.52	6.25	4,2	5.74	6,55	3,8	7.73	29.32	28,4
	±0.18	±0.54	±1.6	±0.62	±0.83	±1.1	±0.74	±0.31	±0.5	±0.08	±0.93	±0,3	±0.03	±0.31	±4,0
**700**	40.23	26.07	23,3	13.47	25.58	22,9	70.20	10.01	9,6	12.88	5.82	6,6	4.06	40.06	36,0
	±0.00	±5.03	±1.1	±0.37	±4.60	±1.2	±0.71	±0.33	±0.2	±0.26	±0.35	±1,0	±0.07	±2.46	±1,7
**701**	43.85	9.32	11,9	9.94	18.84	19,7	51.17	6.45	8,1	7.16	5.56	6,5	2.23	38.84	52,3
	±1.25	±1.72	±2.0	±0.02	±1.89	±1.3	±0.89	±0.95	±0.4	±0.20	±0.69	±1,3	±0.01	±8.60	±1,9
**705**	33.40	30.40	30,3	7.65	45.46	53,2	54.69	16.49	16,4	4.48	20.44	25,7	8.39	66.32	89,9
	±1.61	±2.62	±3.3	±0.28	±4.58	±5.1	±3.56	±2.22	±1.6	±0.30	±2.44	±2,1	±0.12	±1.44	±3,5
**720**	25.76	19.79	26,8	5.57	40.66	67,3	62.34	10.43	12,4	5.08	11.21	18,5	16.31	67.27	98,0
	±1.16	±4.32	±4.0	±0.07	±4.75	±1.8	±1.81	±1.22	±1.0	±0.13	±2.07	±1,8	±0.17	±2.48	±7,4
**723**	20.69	35.14	41,6	5.66	28.79	38,3	61.43	8.96	11,7	5.05	8.27	14,3	18.78	73.63	130,4
	±0.83	±2.61	±7.6	±0.04	±1.97	±5.6	±0.28	±0.38	±2.2	±0.04	±0.63	±1,9	±0.16	±1.57	±7,7
**865**	40.44	1.25	2,3	5.56	n.d.	3,9	51.44	1.40	2,5	3.18	n.d.	n.d.	2.37	17.75	22,2
	±0.38	±0.08	±0.1	±0.31		±0.3	±0.02	±0.28	±0.5	±0.02			±0.03	±0.94	±1,2

n.d. = not detectable

The two teas with the highest theanine concentrations (16.3 mg/g and 18.8 mg/g) are remarkable because the tea with the third highest theanine content has only 10.2 mg theanine/g. These two teas are the only shaded teas among the samples (Table 1). Shading is known to increase theanine levels in tea leaves [28,29].

The extraction efficiency, *i.e.*, how much of a compound is extracted compared to the total amount in the tea leaves (Table 2), follows the same trends as mentioned in section 3.1. The non-galloylated flavanols are extracted easier resulting in extraction efficiencies of 26 % and 28 % (medians) for EGC and EC, respectively, in the first infusion. The galloylated flavanols show extraction efficiencies of 10 % and 14 % (medians) for EGCg and ECg, respectively, in the first infusion. Theanine as the most polar compound shows an extraction efficiency of 40 % (median) in the first infusion. Thus, the major part of the flavanols and theanine is still in the tea leaves after the first infusion. This is why the flavanols can be found in considerable quantities in the second infusion, *i.e.,* especially the galloylated flavanols EGCg and ECg, while the non-galloylated flavanols EGC and EC are mainly found in higher concentrations in the first infusion; theanine is dominant in the first infusion mostly by factor 1.4 to 2.2. Similar values for the extraction efficiency of the flavanols have been published previously [8,18,30]. One tea is out of the usual range: tea 865 showed a markedly low extraction efficiency, which is caused by its leaf size and structure (see 3.1).

The extraction efficiency of the second infusion is calculated by dividing the amount extracted in the second infusion by the remaining amount in the leaves after the first infusion. In more than half of the samples, it is higher than the extraction efficiency of the first infusion (see Supplementary data). The mean extraction efficiency of the second infusion is 1.2–1.4 times higher than the mean extraction efficiency of the first infusion for the flavanols. In the case of theanine, it is higher by a factor of 1.9. Hence, the second infusion is conditioned by two opposing mechanisms: the tea leaves contain a lower percentage of the phytochemicals, which is obvious. However, our data show that the extraction efficiency is higher at the second infusion, probably because the leaf structure is already soaked with water and more permeable. The consequence is that the second infusion contains similar concentrations of EGCg and ECg and slightly diminished concentrations of EC, EGC and caffeine (medians 18 %–26 % less), as already mentioned in section 3.1. Only the most polar compound considered, theanine, has a markedly smaller percentage of 58 % (median) in the second infusion.

## 4 Conclusion – from the consumer's perspective

Does the second infusion of green leaf tea still contain relevant amounts of caffeine and possibly health-related compounds like flavanols and theanine? The short answer is: yes. The long answer is: in most cases, yes. It depends on the tea qualities and the phytochemicals in consideration. In most cases, the EGCg dose is similar in both infusions but, in some cases, lower, although still considerable. Theanine, instead, is found in lesser amounts in the second infusion. But even in the worst case of this study, preparing and drinking the second infusion increases the dose of theanine by a factor of ca. 1.5 compared to drinking only the first infusion. Anyhow, it is not known which doses of EGCg, the other flavanols and theanine are necessary to get a specific health effect. This is different for caffeine: the health claims about caffeine that the EFSA positively reviewed require 3–4 mg caffeine per kg bodyweight [1], equal to 180–240 mg caffeine for a 60-kg person. This is hard to reach by drinking tea: roughly two to six liter of the teas in this study would have to be drunk. If someone aims to maximize the phytochemical intake, the main factor is choosing the right tea. Additionally, the steeping temperature and time could be increased for higher extraction efficiency, but this leads to unpleasantly bitter brews; therefore, the brewing recommendations on the product label were followed to get a tasty tea.

Having said this, it's obvious that with the same amount of tea leaves, a relevantly higher intake of phytochemicals is possible by preparing a second infusion. To put it the other way around: the same amount of phytochemicals intake can be reached with fewer tea leaves by preparing a second infusion instead of brewing another cup with fresh tea leaves. This increases the sustainability of tea consumption.

## CRediT authorship contribution statement

**Benno F. Zimmermann:** Conceptualization, Formal analysis, Methodology, Supervision, Validation, Visualization, Writing – original draft, Writing – review & editing. **Lilli Drees:** Investigation, Methodology, Validation, Visualization, Writing – review & editing.

## Declaration of competing interest

The authors declare that they have no known competing financial interests or personal relationships that could have appeared to influence the work reported in this paper.
