# Supplementary material for: Let's have another cup of green tea!
Source: Heliyon. 2025 Feb 15;11(4):e42651. doi: 10.1016/j.heliyon.2025.e42651 (PMC11908554; doi:10.1016/j.heliyon.2025.e42651)
Supplement: Multimedia component 1 [file mmc1.pdf]

EGC Content in the Infusions

Tea sample	n	First infusion [mg/portion]	First infusion [mg/L]	Extracted percentage in the first infusion[%]	n	Second infusion [mg/portion]	Second infusion [mg/L]	Extracted percentage in the second infusion [%]
**293**	2	18.39 ± 1.07	73.56 ± 4.28	25.53 ± 2.66	2	10.10 ± 1.86	40.40 ± 7.44	14.28 ± 2.78
**310**	2	21.02 ± 2.87	84.08 ± 11.48	27.21 ± 3.61	2	16.70 ± 0.61	66.80 ± 2.44	21.96 ± 0.97
**313**	2	32.90 ± 2.12	131.60 ± 8.48	34.01 ± 3.13	2	27.23 ± 4.69	80.24 ± 3.48	27.19 ± 4.82
**355**	4	29.66 ± 4.63	118.64 ± 18.52	32.73 ± 4.98	2	20.06 ± 0.87	80.24 ± 3.48	21.98 ± 1.14
**510**	4	8.20 ± 1.28	32.80 ± 5.12	13.32 ± 2.50	2	5.79 ± 0.98	23.16 ± 3.92	10.10 ± 2.03
**516**	4	10.09 ± 0.86	40.36 ± 3.44	18.20 ± 1.92	2	6.20 ± 1.14	24.80 ± 4.56	11.27 ± 2.06
**590**	4	32.02 ± 4.06	128.08 ± 1.8	30.57 ± 5.02	2	24.96 ± 5.07	99.84 ± 20.28	21.62 ± 4.11
**591**	4	46.08 ± 0.45	184.32 ± 1.8	39.99 ± 1.42	2	34.98 ± 5.90	139.92 ± 23.60	30.14 ± 4.91
**596**	2	13.64 ± 0.15	54.56 ± 0.60	18.53 ± 1.72	2	8.54 ± 2.12	34.16 ± 8.48	11.72 ± 3.01
**597**	2	15.17 ± 1.80	60.68 ± 7.2	27.37 ± 3.50	2	9.63 ± 2.23	38.28 ± 8.92	17.56 ± 4.61
**665**	2	17.61 ± 1.05	70.44 ± 4.20	13.60 ± 0.54	2	14.57 ± 2.71	58.28 ± 10.84	8.39 ± 1.41
**700**	2	28.89 ± 5.90	115.56 ± 23.60	26.07 ± 5.03	2	20.82 ± 1.05	83.28 ± 4.2	18.75 ± 0.86
**701**	2	11.16 ± 2.03	44.64 ± 8.12	9.32 ± 1.72	2	12.97 ± 2.18	51.88 ± 8.72	10.66 ± 1.76
**705**	4	33.22 ± 3.26	132.88 ± 13.04	30.40 ± 2.62	2	21.11 ± 1.19	84.44 ± 4.76	21.45 ± 2.34
**720**	4	22.19 ± 2.78	88.76 ± 11.12	19.79 ± 4.32	2	15.21 ± 1.84	60.84 ± 7.36	21.84 ± 3.27
**723**	4	18.67 ± 1.01	74.68 ± 4.04	35.14 ± 2.61	2	15.34 ± 2.70	61.36 ± 10.80	26.47 ± 4.83
**865**	2	1.54 ± 0.10	6.16 ± 0.4	1.25 ± 0.08	2	2.81 ± 0.08	11.24 ± 0.32	2.29 ± 0.13
**Range**		1.54 ±0.10 – 46.08 ±0.45	6.16 ±0.4 – 184.32 ±1.8	1.25 ±0.08 – 39.99 ±1.42		2.81 ±0.08 – 34.98 ±5.90	11.24 ±0.32 – 139.9±23.6	2.29 ±0.13 – 30.14 ±4.91

1 portion = 250 ml; n = number of replicates; all results are presented as mean ± standard deviation

EC content in the infusions

Tea sample	n	First infusion [mg/portion]	First infusion [mg/L]	Extracted percentage in the first infusion[%]	n	Second infusion [mg/portion]	Second infusion [mg/L]	Extracted percentage in the second infusion [%]
**293**	2	4.96 ± 0.45	19.84 ± 1.80	21.17 ± 2.42	2	3.25 ± 0.64	13.00 ± 2.56	14.13 ± 2.80
**310**	2	4.03 ± 0.52	16.12 ± 2.08	25.34 ± 3,29	2	2.95 ± 0.12	11.80 ± 0.48	18.85 ± 1.05
**313**	2	9.26 ± 0.41	37.04 ± 1.64	30.70 ± 1.26	2	8.34 ± 1.42	33.36 ± 5.68	26.99 ± 4.75
**355**	4	10.27 ± 0.80	41.08 ± 3.20	30.08 ± 2.74	2	7.58 ± 0.59	30.32 ± 2.36	22.04 ± 2.24
**510**	4	3.18 ± 0.47	12.72 ± 1.88	25.19 ± 4.36	2	1.97 ± 0.10	7.88 ± 0.40	16.04 ± 1.39
**516**	4	2.44 ± 0.26	9.76 ± 1.04	27.80 ± 2.56	2	1.48 ± 0.05	5.92 ± 0.20	16.99 ± 0.49
**590**	4	9.10 ± 1.29	36.40 ± 5.16	31.35 ± 4.70	2	8.05 ± 1.16	32.20 ± 4.64	27.15 ± 3.55
**591**	4	11.27 ± 0.43	45.08 ± 1.72	40.01 ± 2.56	2	8.54 ± 0.99	34.16 ± 3.92	30.12 ± 3.89
**596**	2	8.92 ± 0.74	35.68 ± 2.96	16.49 ± 1.87	2	5.86 ± 0.66	23.44 ± 2.64	10.98 ± 1.46
**597**	2	13.49 ± 1.60	53.96 ± 6.40	28.50 ± 3.67	2	9.58 ± 1.35	38.32 ± 5.4	20.44 ± 3.54
**655**	2	4.98 ± 0.27	19.92 ± 1.08	12.87 ± 0.83	2	3.41 ± 0.37	13.64 ± 1.48	8.95 ± 0.94
**700**	2	9.49 ± 1.80	37.96 ± 7.20	25.58 ± 4.60	2	6.88 ± 0.48	27.52 ± 1.92	18.08 ± 0.93
**701**	2	5.16 ± 0.60	20.64 ± 2.40	18.84 ± 1.89	2	4.37 ± 0.91	17.48 ± 3.64	15.83 ± 1.05
**705**	4	10.53 ± 1.08	42.12 ± 4.32	45.46 ± 4.58	2	6.66 ± 0.86	26.64 ± 3.44	29.45 ± 2.80
**720**	4	6.19 ± 0.62	24.76 ± 2.48	40.66 ± 4.75	2	6.12 ± 0.28	24.48 ± 1.12	40.55 ± 1.06
**723**	4	4.40 ± 0.35	17.60 ± 1.40	28.79 ± 1.97	2	4.24 ± 0.60	16.96 ± 2.40	26.73 ± 3.88
**865**	2	n. d.	n. d.	n. d.	2	0.65 ± 0.02	2.60 ± 0.08	3.86 ± 0.31
**Range**		n. d. – 13.49 ±1.60	n. d. – 53.96 ±6.40	n. d. – 45,46 ±4,58		0.65 ±0.02 – 9.58 ±1.35	2.60 ±0.08 – 38.32 ±5.4	3.86 ±0.31 – 40.55 ±1.06

1 portion = 250 ml; n = number of replicates; n. d. = not detected; all results are presented as mean ± standard deviation

EGCg content in the infusions

Tea sample	n	First infusion [mg/portion]	First infusion [mg/L]	Extracted percentage in the first infusion [%]	n	Second infusion [mg/portion]	Second infusion [mg/L]	Extracted percentage in the second infusion [%]
**293**	2	24.92 ± 3.40	99.68 ± 13.60	13.50 ± 1.95	2	18.54 ± 1.31	74.16 ± 5.24	10.24 ± 0.96
**310**	2	28.08 ± 3.96	112.32 ± 15.84	11.64 ± 1.58	2	27.06 ± 0.62	108.24 ± 2.48	11.39 ± 0.34
**313**	2	50.99 ± 0.98	203.96 ± 3.92	17.05 ± 0.82	2	60.30 ± 8.79	241.20 ± 35.16	19.68 ± 3.04
**355**	4	28.55 ± 2.83	114.20 ± 11.32	13.11 ± 1.16	2	25.99 ± 0.16	103.96 ± 0.64	11.85 ± 0.43
**510**	4	14.63 ± 1.95	58.52 ± 7.80	7.53 ± 1.07	2	15.43 ± 1.31	61.72 ± 5.24	8.18 ± 0.97
**516**	4	12.08 ± 0.94	48.32 ± 3.76	4.75 ± 0.32	2	10.66 ± 0.75	42.64 ± 3.00	4.23 ± 0.33
**590**	4	24.35 ± 3.84	97.40 ± 15.36	13.10 ± 2.21	2	25.96 ± 2.76	103.84 ± 11.04	13.67 ± 1.27
**591**	4	25.32 ± 1.48	101.28 ± 5.92	15.35 ± 0.89	2	26.40 ± 2.47	105.60 ± 9.88	15.89 ± 1.53
**596**	2	15.98 ± 1.27	63.92 ± 5.08	9.17 ± 1.13	2	12.02 ± 1.95	48.08 ± 7.8	6.99 ± 1.27
**597**	2	22.73 ± 1.67	90.92 ± 6.68	14.01 ± 1.30	2	16.58 ± 2.25	66.32 ± 9.00	10.32 ± 1.78
**665**	2	15.49 ± 0.80	61.96 ± 3.20	6.25 ± 0.31	2	9.69 ± 1.31	38.76 ± 5.24	4.02 ± 0.47
**700**	2	19.22 ± 0.62	76.88 ± 2.48	10.01 ± 0.33	2	18.23 ± 1.76	72.92 ± 7.04	9.55 ± 0.23
**701**	2	9.00 ± 1.32	36.00 ± 5.28	6.45 ± 0.95	2	10.69 ± 0.59	42.76 ± 2.36	7.53 ± 0.40
**705**	4	27.30 ± 3.31	109.20 ± 13.24	16.49 ± 2.22	2	22.41 ± 0.70	89.64 ± 2.80	13.90 ± 1.36
**720**	4	17.80 ± 1.86	71.20 ± 7.44	10.43 ± 1.22	2	19.08 ± 0.94	76.32 ± 3.76	11.31 ± 0.87
**723**	4	14.85 ± 0.84	59.40 ± 3.36	8.96 ± 0.38	2	17.98 ± 3.36	71.92 ± 13.44	10.45 ± 1.98
**865**	2	2.19 ± 0.44	8.76 ± 1.76	1.40 ± 0.28	2	3.80 ± 0.62	15.20 ± 1.76	2.44 ± 0.46
**Range**		2.19 ± 0.44 – 50.99 ± 0.98	8,76 ± 1,76 – 204.0 ±3.92	1.40 ± 0.28 – 17.05 ± 0.82		3.80 ± 0.62 – 60.30 ± 8.79	15.20 ±1.76 – 241.2 ±35.2	2.44 ±0.46 – 19.68 ±3.04

1 portion = 250 ml; n = number of replicates; all results are presented as mean ± standard deviation

ECg content in the infusions

Tea sample	n	First infusion [mg/portion]	First infusion [mg/L]	Extracted percentage in the first infusion [%]	n	Second infusion [mg/portion]	Second infusion [mg/L]	Extracted percentage in the second infusion [%]
**293**	2	3.76 ± 010	15.04 ± 0.40	17.28 ± 1.39	2	3.31 ± 0.09	13.24 ± 0.36	15.73 ± 1.26
**310**	2	5.29 ± 0.41	21.16 ± 1.64	11.47 ± 0.85	2	5.11 ± 0.52	20.44 ± 2.08	11.25 ± 1.12
**313**	2	11.22 ± 0.28	44.88 ± 1.12	17.16 ± 1.09	2	13.83 ± 1.32	55.32 ± 5.28	20.62 ± 2.18
**355**	4	7.37 ± 0.49	29.48 ± 1.96	24.31 ± 1.74	2	6.85 ± 0.43	27.40 ± 1.72	22.41 ± 1.46
**510**	4	2.27 ± 0.31	9.08 ± 1.24	12.29 ± 1.64	2	2.37 ± 0.19	9.48 ± 0.76	13.25 ± 0.74
**516**	4	1.04 ± 0.04	4.16 ± 0.16	4.87 ± 0.16	2	1.20 ± 0.12	4.80 ± 0.48	5.38 ± 0.63
**590**	4	2.64 ± 0.41	10.56 ± 16.64	16.58 ± 2.50	2	3.66 ± 0.16	14.64 ± 0.64	22.68 ± 0.68
**591**	4	3.15 ± 0.13	12.60 ± 0.52	22.02 ± 1.14	2	3.38 ± 0.32	13.52 ± 1.28	23.47 ± 2.41
**596**	2	6.87 ± 0.75	27.48 ± 3.00	15.76 ± 2.10	2	5.61 ± 0.54	2.16 ± 2.16	13.04 ± 1.58
**597**	2	13.62 ± 1.41	54.48 ± 5.64	27.57 ± 3.13	2	11.66 ± 1.05	46.64 ± 4.2	23.81 ± 2.91
**665**	2	1.22 ± 0.19	4.88 ± 0.76	6.55 ± 0.93	2	0.66 ± 0.07	2.64 ± 0.28	3.57 ± 0.32
**700**	2	2.06 ± 0.15	8.24 ± 0.6	5.82 ± 0.35	2	2.41 ± 0.17	9.64 ± 0.68	8.27 ± 1.31
**701**	2	1.09 ± 0.14	4.36 ± 0.56	5.56 ± 0.69	2	1.20 ± 0.25	4.80 ± 1.00	6.05 ± 1.24
**705**	4	2.76 ± 0.23	11.04 ± 0.92	20.44 ± 2.44	2	2.75 ± 0.25	11.00 ± 1.00	20.79 ± 1.72
**720**	4	1.56 ± 0.27	6.24 ± 1.08	11.21 ± 2.07	2	2.29 ± 0.27	9.16 ± 1.08	16.63 ± 1.66
**723**	4	1.13 ± 0.10	4.52 ± 0.40	8.27 ± 0.63	2	1.82 ± 0.23	7.28 ± 0.92	12.85 ± 1.68
**865**	2	n. d.	n. d.	n. d.	2	n. d.	n. d.	n. d.
**Range**		n. d. – 13.62 ±1.41	n. d. – 54.48 ±5.64	n. d. – 27.57 ±3.13		n. d. – 13.83 ±1.32	n. d. – 55.32 ±5.28	n. d. – 23.81 ±2.91

1 portion = 250 ml; n = number of replicates; n. d. = not detected; all results are presented as mean ± standard deviation

Theanine content in the infusions

Tea sample	n	First infusion [mg/portion]	First infusion [mg/L]	Extracted percentage in the first infusion [%]	n	Second infusion [mg/portion]	Second infusion [mg/L]	Extracted percentage in the second infusion [%]
**293**	2	3.83 ± 0.38	15.32 ± 1.52	29.46 ± 3.12	2	2.47 ± 0.20	9.88 ± 0.80	19.56 ± 1.87
**310**	2	8.50 ± 0.21	34.00 ± 0.84	39.39 ± 1.12	2	5.39 ± 0.16	21.56 ± 0.64	25.08 ± 1.65
**313**	2	6.99 ± 0.30	27.96 ± 1.20	52.70 ± 2.47	2	4.04 ± 0.40	16.16 ± 1.60	30.29 ± 2.08
**355**	2	2.92 ± 0.03	11.68 ± 0.12	44.48 ± 0.73	2	1.69 ± 0.18	6.76 ± 0.72	25.65 ± 2.73
**510**	2	2.77 ± 0.23	11.08 ± 0.92	36.02 ± 2.76	2	1.89 ± 0.17	7.56 ± 0.68	24.83 ± 2.01
**516**	2	10.11 ± 1.12	40.44 ± 4.48	33.19 ± 4.12	2	5.84 ± 0.20	23.36 ± 0.80	19.16 ± 0.89
**590**	2	5.98 ± 0.53	23.92 ± 2.12	64.62 ± 5.41	2	3.12 ± 0.05	12.48 ± 0.20	33.88 ± 0.53
**591**	2	4.57 ± 0.01	18.28 ± 0.04	69.07 ± 1.35	2	2.10 ± 0.02	8.40 ± 0.08	32.41 ± 0.85
**596**	2	6.11 ± 0.30	24.44 ± 1.2	28.79 ± 1.06	2	3.15 ± 0.02	12.6 ± 0.08	18.86 ± 0.37
**597**	2	7.31 ± 0.17	29.24 ± 0.68	41.62 ± 1.72	2	4.39 ± 0.55	17.56 ± 2.20	25.83 ± 2.95
**665**	2	7.24 ± 0.13	28.96 ± 0.52	29.32 ± 0.31	2	5.04 ± 0.75	20.16 ± 3.00	20.05 ± 2.83
**700**	2	4.52 ± 0.24	18.08 ± 0.96	40.06 ± 2.46	2	2.63 ± 0.14	10.52 ± 0.56	23.28 ± 1.08
**701**	2	2.45 ± 0.52	9.38 ± 2.09	38.84 ± 8.60	2	1.96 ± 0.09	7.85 ± 0.37	32.07 ± 1.18
**705**	2	16.42 ± 0.06	65.68 ± 0.24	66.32 ± 1.44	2	7.62 ± 1.25	30.48 ± 5.00	30.71 ± 1.19
**720**	2	29.85 ± 1.40	119.40 ± 5.60	67.27 ± 2.48	2	14.39 ± 0.86	57.56 ± 3.44	32.28 ± 2.44
**723**	2	38.30 ± 1.23	153.20 ± 4.92	73.63 ± 1.57	2	17.76 ± 0.82	71.04 ± 3.28	33.62 ± 1.99
**865**	2	1.26 ± 0.08	5.04 ± 0.32	17.75 ± 0.94	2	1.30 ± 0.06	5.20 ± 0.24	18.30 ± 0.97
**Range**		1.26 ± 0.08 – 38.3 ± 1.23	5.04 ± 0.32 – 153.2 ±4.92	17.75 ± 0.94 – 73.63 ±1.57		1.3 ± 0.06 – 17.76 ±0.82	5.2 ± 0.24 – 71.04 ±3.28	18.30 ± 0.97 – 33.88 ± 0.53

1 portion = 250 ml; n = number of replicates; all results are presented as mean ± standard deviation

Caffeine content in the infusions

Tea sample	n	First infusion [mg/portion]	First infusion [mg/L]	n	Second infusion [mg/portion]	Second infusion [mg/L]
**293**	2	21.78 ± 3.36	87.12 ± 13.44	2	14.12 ± 0.43	56.48 ± 1.72
**310**	2	23.51 ± 2.30	94.04 ± 9.20	2	18.32 ± 5.77	73.28 ± 23.08
**313**	2	28.54 ± 0.80	114.16 ± 3.20	2	29.89 ± 2.79	119.56 ± 11.16
**355**	4	20.80 ± 1.47	83.20 ± 5.88	2	16.44 ± 1.38	65.76 ± 5.52
**510**	4	15.47 ± 1.34	61.88 ± 5.36	2	14.49 ± 0.32	57.96 ± 1.28
**516**	4	20.16 ± 2.64	80.64 ± 10.56	2	17.69 ± 1.85	70.76 ± 7.40
**590**	4	22.73 ± 3.42	90.92 ± 13.68	2	21.37 ± 1.78	85.48 ± 7.12
**591**	4	28.70 ± 1.74	114.80 ± 6.96	2	20.67 ± 2.11	82.68 ± 8.44
**596**	2	14.42 ± 1.08	57.68 ± 4.32	2	10.90 ± 0.84	43.60 ± 3.36
**597**	2	28.59 ± 1.25	114.36 ± 5.00	2	19.33 ± 1.08	77.32 ± 4.32
**665**	2	17.18 ± 1.33	68.72 ± 5.32	2	14.23 ± 0.06	56.92 ± 0.24
**700**	2	22.33 ± 1.19	89.32 ± 4.76	2	17.42 ± 2.29	69.68 ± 9.16
**701**	2	10.65 ± 1.13	42.60 ± 4.52	2	8.98 ± 1.26	35.92 ± 5.04
**705**	4	32.03 ± 5.60	128.12 ± 11.92	2	20.01 ± 1.56	80.04 ± 6.24
**720**	4	28.16 ± 2.98	112.64 ± 11.92	2	27.35 ± 2.82	109.40 ± 11.28
**723**	4	28.99 ± 1.68	115.96 ± 6.72	2	24.39 ± 4.52	97.56 ± 18.08
**865**	2	5.24 ± 0.84	20.96 ± 3.36	2	10,04 ± 0,87	40,16 ± 3,48
**Range**		5.24 ± 0.84 – 32.03 ± 5.60	20.96 ± 3.36 – 128.12 ± 11.92		8,98 ± 1,26 – 29,89 ± 2,79	35,92 ± 5,04 – 119,56 ± 11,16

1 portion = 250 ml; n = number of replicates; all results are presented as mean ± standard deviation
